# Implementing a new multidisciplinary, remote, dementia staff training program for Veterans affairs nursing homes

**DOI:** 10.1186/s12913-024-11464-4

**Published:** 2024-10-03

**Authors:** Nikita R. Shirsat, Jennifer Ann Lee, Catherine Pham, Matthew J. Miller, Margaret A. Chesney, Francesca M. Nicosia, Linda Chao, Deborah E. Barnes

**Affiliations:** 1 San Francisco VA Health Care System, San Francisco, CA USA; 2 University of California, San Francisco, San Francisco, CA USA

**Keywords:** Dementia, Nursing homes, Quality of life, Exercise, Movement therapy, PDSA cycles

## Abstract

**Background:**

Preventing Loss of Independence through Exercise (PLIÉ) is a group program for people living with dementia that combines movements to support daily function with present moment body awareness and social engagement that has been found to have physical, emotional, social, and cognitive benefits. The goal of this study was to develop and refine a PLIÉ remote training program for interdisciplinary Veterans Affairs (VA) nursing home staff members also known as community living center (CLC) staff.

**Methods:**

This pre-implementation study used iterative Plan-Do-Study-Act (PDSA) cycles. The 10-week PDSA cycles occurred from June to September 2021 at 2 VA nursing home sites. Remote training was delivered via Microsoft Teams and included 1-hour live-streamed weekly didactic sessions (nursing staff with PLIÉ instructor) focused on PLIÉ principles and 1-hour weekly live-streamed experiential sessions for staff to apply PLIÉ principles with residents. We administered weekly feedback surveys to iteratively refine the training process.

**Results:**

14 staff members participated (5 recreation therapists, 3 social workers, 2 registered nurses, 2 chaplains, 1 psychologist, and 1 speech pathologist). The experiential sessions were rated as most helpful overall. Key PDSA refinements included: (1) creating 10-minute video recording summaries to support learning, particularly for those unable to attend live training sessions due to clinical schedules; and (2) incorporating self-reflection and goal setting to support staff incorporation of PLIÉ principles into routine care and personal life. These refinements resulted in increased use of PLIÉ principles with the residents from 67 to 89% of the staff participants. 100% of regular attendees (11/11) rated their overall satisfaction with remote training as “very good” or “excellent.”

**Conclusions:**

It was feasible to train interdisciplinary CLC staff participants to deliver an integrative group movement program for residents with dementia remotely. PDSA cycles supported refinement of the training process and improved uptake. A larger study of PLIÉ remote CLC staff training is needed to assess outcomes on residents and quality of care.

**Supplementary Information:**

The online version contains supplementary material available at 10.1186/s12913-024-11464-4.

## Background

There are more than 6 million Americans living with Alzheimer’s disease and related dementias, and nearly half a million of American Veterans are estimated to have Alzheimer’s [1]. Many Veterans have comorbid conditions such as posttraumatic stress disorder, traumatic brain injury, and depression that increase the risk of dementia, behavioral issues, and complicate their care. The Veterans Health Administration (VHA) operates more than 130 skilled nursing facilities, called Community Living Centers (CLCs), where half of the 40,000 Veterans served annually have dementia or neurocognitive disorders. In many cases, CLCs become the primary source of care for Veterans with dementia and comorbid physical and mental health conditions.

Non-pharmacologic interventions have many benefits for people living with dementia. For example, physical activity has been shown to improve physical function; [2] cognitive/social stimulation to improve cognitive function and quality of life; [3] and music therapy to improve mood and behavioral outcomes [4]. However, most non-pharmacologic programs in nursing home settings tend to target one domain at a time rather than using a multi-domain approach. In addition, few of these programs have been adapted to address the complex physical, neurocognitive, and mental health needs of Veterans in the CLC setting such as traumatic brain injuries, substance use disorders and post-traumatic stress disorder (PTSD) [5]. Moreover, CLC staff rarely receive specialized training in how to deliver evidence-based, non-pharmacological interventions for residents with dementia.

Over the past decade, we have systematically developed and tested the Preventing Loss of Independence through Exercise (PLIÉ) program – an integrative, multi-domain, structured group movement program for people across the spectrum of cognitive decline [6–11]. PLIÉ is a gentle, non-aerobic program targeting abilities and neural mechanisms that are relatively preserved in people with dementia. PLIÉ instructors are taught to utilize 8 guiding principles that are woven into the classes: using breath and body awareness to orient in the present moment; creating an environment to support joy and ease; moving and talking slowly to give residents time to process and respond; using movement sequences to help residents with dementia maintain function; using music and rhythm to support positive emotions; treating residents with patience, respect, and dignity; supporting social engagement and emotional connection between residents; and building community between residents, staff and family members.

Veterans have been heavily involved in development and testing of PLIÉ since its inception. The original PLIÉ program was developed for and tested in the adult day setting with funding from the VA [8, 9]. Study sites were adult day centers in the San Francisco Bay Area that contracted with the San Francisco VA (SFVA), and Veterans were prioritized for enrollment. In 2018, we received a VA Innovator’s Award in which we adapted PLIÉ for VHA CLCs based on stakeholder input. Key adaptations included larger class sizes, inclusion of people in wheelchairs and with behavioral challenges, inclusion of family and staff members, and incorporation of movement sequences for a more physically impaired population [10, 12]. We then performed a clinical demonstration project in which we trained two clinical champions at the SFVA CLC to lead PLIÉ classes using an in-person “experiential” process. The training took place over 3 months. For 12 weeks (24 sessions), champions participated in two 1-hour group classes each week with residents led by a senior PLIÉ instructor. Each week’s classes were preceded by the senior PLIÉ instructor sharing the teaching plan for the week, and each class was followed by debrief with the clinical champions and the senior PLIÉ instructor. In addition, the champions received an instructor training manual. After the 12-week training, staff were responsible for leading PLIÉ classes independently with periodic check-ins.

We evaluated implementation of staff-led classes based on reach and effectiveness 12 months after training completion and found that champions had continued PLIÉ-CLC classes with an average of 22 participants (14 residents, 4 staff members, 2 family members, 2 instructors) per class [11]. Participants reported social, physical, and cognitive benefits, and classes were highly rated by participants, staff, and family. Although the 12-week, in-person PLIÉ-CLC training in the San Francisco CLC appeared to be highly effective, we recognized that it would be financially and logistically challenging to disseminate this training model across VHA CLCs nationally. Therefore, the goal of the current study was to pilot and refine a PLIÉ remote training program for interdisciplinary CLC staff participants by assessing staff participants’ outcomes such as their confidence levels and their usage of PLIÉ principles.

## Methods

In this pre-implementation study, we used iterative Plan-Do-Study-Act (PDSA) cycles to inform refinement of the remote PLIÉ staff training program. PDSA is commonly used in healthcare settings as a tool to systematically assess the impact of change by planning (Plan), implementing (Do), observing and learning from the results (Study), and acting based on results to make practice modifications (Act) [13–16]. We used an adapted version of the PDSA cycle framework by assessing a new question each week to obtain feedback on the training program and process.

### Setting & participants

The study took place remotely and synchronously at two CLCs in Northern California and included the original clinical demonstration site, which wanted to train new instructors, and a second site that had not previously implemented PLIÉ. At each site, one or more clinical champions volunteered to coordinate the trainings including recruiting staff participants and identifying residents. Each clinical champion, staff participant, resident, and legally authorized representative LAR (for CLC residents without capacity) provided informed consent to participate in this research. Thus, our target population was the CLC staff. Demographic data that was captured included participants’ roles at the CLCs, gender and race. All study activities were reviewed and approved by the Veterans Affairs Central Institutional Review Board (IRB# 1612624-6). The authors of this manuscript have no financial or personal conflicts of interest.

### Pre-training planning activities

To generate support for piloting remote PLIÉ-CLC training, the research team first presented an overview of PLIÉ and the training program to facility leadership, managers, and staff. The senior instructor (JAL), clinical research coordinator (CP), and champions at each site met weekly for four months for pre-training planning. We conducted a range of pre-training activities to prepare for smooth implementation of the training. Pre-training activities included but were not limited to assessing technology needs, scheduling, contingency planning for COVID quarantines and ordering and shipping supplies such as instructor training manuals and sensory engagement tools. The research team also facilitated iPad access by assisting the VA staff in obtaining VA-provider iPads. The staff participants used the iPads to partake in the PLIÉ experiential sessions from residents’ rooms.

### Training structure

The training included an orientation session, nine didactic sessions, and ten experiential sessions that immediately follow the didactic. The didactic sessions consisted of weekly 1-hour, live-streamed, interactive lessons led by the senior instructor who demonstrated PLIÉ principles and movements through interactive instruction with staff participants and residents. The training was done during the workday, and night shift staff were invited to review video materials from recorded live-streaming didactic and experiential sessions. The research team was able to conduct the training sessions with both CLCs while maintaining COVID-19 safety protocols by using Microsoft Teams, an online, interactive, enterprise platform to host ongoing live training communication, and training materials including surveys, and staff engagement. The PLIÉ program was taught virtually as a group intervention to the staff participants. Since this training was taught during the COVID-19 pandemic, the staff participants taught the PLIÉ concepts in a one-on-one setting to residents to adhere to COVID-19 restrictions on group programming.

### PDSA cycles

We performed weekly PDSA cycles to iteratively refine the content and structure of the remote staff training program. Each week, we identified a goal for refining the training program and defined steps to achieve the goal (Plan). Then, we developed and administered a brief survey to test the implementation of the goal (Do). Survey responses were reviewed by the research team each week (Study). We then applied survey findings to determine the research question for the next PDSA cycle (Act).

### Pre/Post measure of confidence teaching PLIÉ themes

We measured confidence teaching the 8 PLIÉ themes before and after the training on a Likert scale from 0 (no confidence) to 7 (complete confidence).

### Post-training evaluation

At the conclusion of the training program at each site, we administered a survey (Supplementary File 2) that included overall satisfaction ratings and open-ended questions about the impact of the PLIÉ-CLC remote training for both staff members and residents. We also conducted focus group discussions with participants to elicit more detailed feedback about the didactic and experiential sessions and training program materials. Participants from each site were invited to participate in a virtual focus group discussion over Microsoft Teams and facilitated by a member of the research team (FN) who was not directly involved in training activities. Focus groups were recorded, transcribed, and the facilitator also took detailed notes and wrote a summary for each site. One participant was unable to attend the focus group discussion and completed an individual interview. The focus group interview (Supplementary File 2) was conducted with the day staff and night staff separately.

### Analysis

We used descriptive statistics (mean, standard deviation, and proportion) to summarize staff participants’ demographic characteristics, attendance, and quantitative survey data. We used paired t-tests to compare confidence scores before vs. after training for the staff. We used a rapid qualitative analysis process to analyze focus group discussions [17]. First, we created an a priori Microsoft Excel template organized by focus group guide domains and site [18]. After each focus group, we filled out the analysis matrix with key points from detailed notes. Once transcripts were received, another member of the study team reviewed the templates and transcripts for accuracy and identified quotations to support the key takeaways [19]. Subsequent analysis included organizing responses by site into summary documents for facilitators and barriers to the didactic and experiential training sessions.

## Results

A total of 19 staff, including 3 champions, participated in the PLIÉ training. Three participants withdrew due to clinical duties (*N* = 1), shift change (*N* = 1), and personal leave (*N* = 1). We also excluded two participants’ as they did not partake in any of the didactic sessions or complete any surveys. The remaining 14 participants (age range: 27–75 years) completed the 10-week remote training refinement process from June 2021 to September 2021 and were included in our study for analysis (Table 1). Careers for the participants included recreation therapist, social worker, registered nurse, chaplain, speech pathologist and psychologist.

**Table 1:** Demographic characteristics of participants (*N* = 14)

Characteristic	*n* (%)
Role at the CLC	
Recreation Therapist	5 (35.7)
Social Worker	3 (21.4)
Registered Nurse	2 (14.3)
Chaplain	2 (14.3)
Speech Pathologist	1 (7.1)
Psychologist	1 (7.1)
Gender	
Female	11 (78.6)
Male	3 (21.4)
Race^1^	
White	7 (50.0)
Asian	5 (35.7)
Native Hawaiian or Other Pacific Islander	3 (21.4)
American Indian or Alaska Native	1 (7.1)

^1^Percentages for race > 100% because 2 individuals identified as more than 1 race (1 was American Indian and Native Hawaiian or Other Pacific Islander; 1 was Asian and Native Hawaiian or Other Pacific Islander)

In terms of the residents who took part in the exercise sessions, the mean age of the 25 individuals was 75 ± 13.2. All 25 identified as male and 13 (52%) identified as White, 8 (32%) as Black or African American, and 3 declined to answer about their race. For ethnicity, 3 (76%) identified as Hispanic/Latino. All of the residents who participated in the group activities in the study had dementia or cognitive impairment, and were able to participate in group activities, meaning they did not have challenging behaviors that would preclude group participation.

### Attendance

Eleven individuals attended the sessions regularly (at least 7 out of the 10 livestream sessions). Three were unable to attend the livestream sessions regularly due to clinical and work schedules, of whom two watched video recordings of the sessions. A total of 8 to 13 individuals attended each session. The range of weekly attendance for didactic sessions and experiential sessions was 64.3–85.7%, and the range for survey completion was 57.1–92.9% of participants (Table 2).

**Table 2:** Summary of Weekly Attendance of participants at Live Sessions (*N* = 14)

Module	Didactic Attendance *n* (%)	Experiential Attendance *n* (%)	Survey Completion *n* (%)
Orientation	12 (85.7)	12 (85.7)	13 (92.9)
1	11 (78.6)	11 (78.6)	13 (92.9)
2	10 (71.4)	10 (71.4)	10 (71.4)
3	9 (64.3)	9 (64.3)	12 (85.7)
4	9 (64.3)	12 (85.7)	12 (85.7)
5	8 (57.1)	9 (64.3)	9 (64.3)
6	9 (64.3)	9 (64.3)	12 (85.7)
7	11 (78.6)	12 (85.7)	9 (64.3)
8	9 (64.3)	9 (64.3)	8 (57.1)
9	10 (71.4)	10 (71.4)	11 (78.6)

### PDSA cycles

Details of the 9 PDSA cycles over the 10-week training period are represented in the Appendix (Supplementary File 1).

Cycle 1. Plan: We tested the use of Microsoft Forms to obtain background information on PLIÉ staff participants. Do: We provided a link to the Forms survey at the end of the Orientation session and emailed the link through Microsoft Teams to those who did not attend the session live. Study: Based on the 13 responses, we determined that Forms was effective for surveying those who attended the live sessions. Act: We determined that alternate survey methods would be necessary for those unable to attend live.

Cycle 2: Plan: We sought feedback on the Module 1 didactic session. Do: We surveyed participants about their likelihood of applying the PLIÉ principles, observations from the sessions, and suggestions for future training sessions. Study: All 11 participants who attended live completed the survey and stated that they were “very likely” or “moderately likely” to use what they learned in their work with residents. Only 2 of the 3 participants who did not attend live completed the survey. Act: We determined that alternative training formats would be needed to support those unable to attend the sessions live.

Cycle 3: Plan: We tested whether creating a 10-minute didactic video recording for Module 2 would encourage trainees who did not attend the live training to review didactic content and complete the feedback survey. Do: We emailed the 10-minute Module 2 didactic video recording and link to the feedback survey to non-live attendees. Study: Two of 4 people who did not attend the live session responded and found the video to be at least “moderately useful”. Act: We determined that the 10-minute video recordings could support participation of those unable to attend live.

Cycle 4: Plan: We assessed which training materials were most helpful and being used for individuals attending live sessions and participating asynchronously. Do: Surveys were tailored to reflect the training experiences of each group. Email and Teams reminders were sent to those who did not complete the survey. Study: 12 participants completed the surveys (9 of 9 live attendees, 3 of 5 non-live participants). All respondents rated the experiential sessions as “very helpful” (*n* = 9) or “moderately helpful” (*n* = 3). Act: We determined that multiple reminders were needed to increase response rates for those not attending live.

Cycle 5: Plan: We determined what PLIÉ principles staff were practicing and in what settings. Do: Surveys included questions about use and impact of PLIÉ themes in the last week. Study: The most common themes used were “Sense & Breathe” and “Go Slow.” Eight of 9 (89%) respondents reported using PLIÉ themes in their personal lives and 6 of 9 (67%) used principles with the Veterans at least once in the past week. Act: We determined that participants needed more examples and instruction to help them integrate PLIÉ into their daily interactions with residents.

Cycle 6: Plan: We incorporated self-reflection to increase use of PLIÉ-themes in daily practice. Do: We distributed a survey asking how staff planned to use PLIÉ in their daily interactions with residents and personal lives. Study: Five of 12 (42%) said they would use elements from the principle “Go Slow” and 5 of 12 (42%) said they would use breathing or body awareness exercises inspired by the principle “Sense & Breathe” with residents. Eight of 12 (67%) said they would consciously breathe throughout the day to center themselves. Act: We tested whether self-reflection increased incorporation of PLIÉ themes in their daily interactions with residents and their personal lives by readministering the survey.

Cycle 7: Plan: We determined whether staff participants increased use of PLIÉ themes following the self-reflection exercise. Do: The survey asked what staff did to use PLIÉ in their daily interactions with residents and in personal lives. Study: Eight of 9 respondents (89%) used PLIÉ in their daily interactions with residents at least once in the past week, of whom five (56%) used PLIÉ themes at least once every few days. Eight of 9 (89%) used PLIÉ in their daily life at least once in the past week: multiple times every day (*n* = 2), once a day (*n* = 1), once every few days (*n* = 4), and once every week (*n* = 1). Seven participants described being mindful of their breath as an example. Act: Asking participants explicitly to think about how they might incorporate PLIÉ principles into their daily interactions with residents increased use of PLIÉ themes at least once a week from 67 to 89%.

Cycle 8: Plan: We gathered information on what resources would be needed to support maintenance of PLIÉ classes after training completion and future certification standards. Do: The survey for this cycle asked participants for their ideas on how to implement PLIÉ classes on an ongoing basis and certify new instructors and resources that might help instructors feel comfortable leading or facilitating PLIÉ classes. Study: Survey respondents reported that individual class teaching plans and a library of short videos would be the most helpful. Recommended certification standards included participating in a 6-to-8-session didactic and experiential program, testing trainees, and leading a class with instructor observation. Act: We will create and share resources that trainees identified as being most helpful and develop a certification test to confirm knowledge of PLIÉ principles for future trainees.

Cycle 9: Plan: We determined whether confidence using PLIÉ principles changed in those who completed the training. Do: We repeated the confidence survey. Study: The average confidence scores for the 10 individuals who completed both the pre- and post-surveys declined slightly from before the training to after the training (from 6.6 ± 0.8 to 6.4 ± 0.8) (Table 3). Confidence score declines were greatest for “Use movement sequences to help residents with dementia maintain function” (p = 0.04) and “Use music and rhythm to support positive emotions” (p = 0.007) (Table 3). Act: Results will be used to inform larger-scale testing of the PLIÉ-CLC remote staff training program.

**Table 3:** Changes in staff participants’ average confidence levels regarding PLIÉ themes throughout the training (*N* = 10)

On a scale from 0 (no confidence) to 10 (complete confidence), please rate your confidence in the following areas	Average pre-confidence	Average post-confidence	*p*-values
Use breathing & body awareness to orient residents to the present moment	6.6 ± 1.1	6.2 ± 0.9	0.60
Create an environment to support joy and ease	6.5 ± 0.7	6.4 + 0.8	0.59
Move and talk slowly to give residents time to process & respond	6.7 ± 0.7	6.7 ± 0.7	1.0
Use movement sequences to help residents with dementia maintain function	6.4 ± 0.8	6.0 ± 1.1	0.037
Use music and rhythm to support positive emotions	6.5 ± 0.8	5.4 ± 1.4	0.007
Treat residents with patience, respect & dignity	6.8 ± 0.6	6.9 ± 0.3	0.34
Support social engagement & emotional connection between residents	6.6 ± 0.7	6.7 ± 0.7	0.34
Build community between residents, staff, and family members	6.6 ± 0.7	6.7 ± 0.7	0.34
**Total Average**	6.6 ± 0.8	6.4 ± 0.8	0.32

### Staff satisfaction survey

100% (11/11) of respondents rated their overall satisfaction with the training as “very good” or “excellent.” Staff reported a variety of benefits in themselves from participating in the PLIÉ program (Table 4). Many expressed that the PLIÉ training encouraged a sense of present-moment body awareness in their lives. For instance, one individual said they “have more awareness to slow down and be in the moment;” another stated that they have become “more mindful with vet[eran] interactions.” Other participants described educational benefits from the PLIÉ training: “I enjoyed the didactic lessons … it was interesting to learn that the paired session where we were able to physically engage with the Veteran in movements together enabled me to see firsthand how mirror neurons actually work.” Another stated that they enjoyed the “collective energy, the learning environment and the prospect of doing something new in patient care.”

Staff participants also reported increased mobility, enjoyment and engagement among the residents during the PLIÉ classes. One staff participant stated, “My patient showed a significant change in his physical movement - from wheeling his [wheel chair] independently to showing an improved ability to perform daily activities thus preventing falls.” Another commented: “The resident I have worked with seems to anticipate the movements (i.e., better able to do them) and seems to anticipate that participating will make him feel good.”

**Table 4:** What staff participants like most about the PLIÉ -CLC staff training program

Main Category	Staff Quotes
Hands-On Experience	“The experiential times w/the Veterans”
Hands-On Experience	“The collective energy, the learning environment and the prospect of doing something new in patient care”
Hands-On Experience	“I enjoyed having the hands on experience. It helped to get a good feel for what was needed and how things should be facilitated.”
Interdisciplinary Team/Community Interactions	“I like the group interaction between staff and Veteran. Community aspect”
Interdisciplinary Team/Community Interactions	“It is using a team concept which is useful.”
Interdisciplinary Team/Community Interactions	“Physical and mental engagement with the team and the patients”
Present-Moment Body Awareness	“Learning new ways to connect with my body to improve well-being, and learning to share those with Veterans.”
Multimodal Learning	“I enjoyed the didactic lessons on Friday, as I felt it not only provided the opportunity to process our experience with the Veteran in our Paired group that week, but provided the opportunity to learn the meaning behind each of the movements implemented. For example, it was interesting to learn that the paired session where we were able to physically engage with the Veteran in movements together enabled me to see first hand how mirror neurons actually work.”

### Post-training debrief results

The debrief focus group discussions from site 1 (*N* = 9) and site 2 (*N* = 7) focused on the didactic and experiential sessions and training program logistics.

#### Didactic Sessions

Participants from both sites shared positive feedback about the senior instructor’s demonstrations of PLIÉ, the multi-site group format, and the video recordings. One participant stated that hearing the experiences of the other site “made [the training] richer.” Participants utilized video recordings to review course material if absent during the live training sessions or to review course material at home with fewer distractions than while at work. Some participants found the 1-hour didactic session was rushed or that they were distracted by competing work responsibilities.

#### Experiential Sessions

Participants from both sites noted the portability and flexibility of using iPads, which allowed them to participate in the PLIÉ experiential sessions from residents’ rooms. One participant stated the iPads allowed them to “still gather and still be separate.” Participants stated that watching the senior instructor’s “modeling of how to engage and uplift the Veterans” and flexibility in responding to residents in the present moment taught them skills that could be translated beyond PLIÉ and exercise.

Barriers to the experiential session included shifting COVID-19 policies, the time constraints of rushing directly from the didactic session, lack of space in residents’ rooms, and confusion about which supplies to prepare each week.

#### Logistical considerations

Logistical barriers included troubleshooting technology and internet connection and including alternate residents when a primary resident participant was unable to attend due to illness, medical appointments, or other reasons.

## Discussion

Our pre-implementation study to pilot and refine a PLIÉ remote training program for interdisciplinary CLC staff participants demonstrated that the program successfully trained multidisciplinary CLC staff members through a live remote training with access to supporting content. The training program also fostered high satisfaction for staff who attended and provided opportunities for their self-care, including encouraging a sense of present-moment body awareness.

Participants’ feedback demonstrated that the live component is important for successful learning. All the participants found the experiential sessions helpful in learning through experiencing the PLIÉ movements and observing the live application of the PLIÉ principles with the residents. For future trainings, we plan to continue to incorporate a live experiential component so individuals can experience the PLIÉ program while learning about PLIÉ in the didactic sessions. One participant who was not able to attend the experiential sessions live still reported learning from watching video recordings of the live experiential sessions.

Consistent with previous studies of the PLIÉ program, this current study suggests that PLIÉ may have beneficial effects on residents and staff participants based on staff reporting [6–9]. For example, the staff noticed improved engagement, enjoyment and physical function among the residents, supporting past PLIÉ studies’ results [6, 8, 9, 11]. Staff also stated the positive effects of participating in the program included improved present-moment body awareness for themselves.

Our results are also consistent with other papers that have demonstrated the success of synchronous and asynchronous staff remote training programs [20, 21]. For example, a 2022 study conducted in 16 nursing homes in the UK determined that a digital version of the WHELD program, which aims to improve the health of nursing home residents, supported by remote coaching has significant benefits for care staff and residents with dementia [20, 21]. This is important because our program is designed as a group activity, and we were able to train staff successfully during the COVID-19 pandemic when group activities were often restricted. Our program was intended to be taught remotely to enable scaling, and fortunately, this virtual training format helped eliminate the risk that the training could cause coronavirus infection transmission to vulnerable patients at nursing homes [21]. One benefit of virtual groups is that participants can join the class from any location, even within the same facility. This makes it feasible to train more staff with minimal disruption to clinical schedules.

One strength of the study was that we used rigorous PDSA cycles to learn and revise the remote staff training program. Weekly team discussions of barriers and facilitators for the training program enabled us to identify and address barriers as well as reinforce facilitators. Before the study, we also incorporated virtual site visits and planning to inform the training process. We also had two sites enrolled in our study: 1 site was familiar with the PLIÉ-CLC program, and the other site had received no prior exposure to it.

A surprising finding was that confidence teaching the PLIÉ principles was higher before, rather than after the training. This may reflect over-confidence at baseline or regression to the mean since baseline confidence levels were very high. The largest decline in confidence was reported for using music and rhythm to support positive emotions. This may reflect technical challenges with using music when teaching remotely. Additionally, two skills that participants learned in our training that represent content that is less likely to be a part of staff general training include “use movement sequences to help residents with dementia maintain function” and “use music and rhythm to support positive emotions”. The decrease in confidence levels of these principles encourages us to focus more on increasing instruction about these topics.

Another one of our study’s strengths was that we offered several virtual options for individuals to attend the training, such as through using a laptop or iPad. Participants could use both didactic and experiential materials as well as video recordings to learn about the PLIÉ principles. These multiple forms of presentation of PLIÉ were intended to reinforce concepts for better understanding of the program.

One limitation to our study was that we had a small sample size of 14 staff participants, only 11 of whom attended regularly. Since this study was done in sites in a limited geographic region, the results may not be generalizable to other nursing homes and Community Living Centers. As with training programs for any innovation within complex health systems, participant attendance was limited by clinical schedules, staff shortages, increased workload. Although we attempted to use alternative strategies, such as 10-minute didactic video recordings, we may need to identify additional strategies to offer education to the participants who cannot attend live sessions, such as hosting session in the evening and providing meals during the session. In addition, we plan to offer continuing education (CE) credits to staff members to encourage more individuals to participate in the program. Another limitation was that we did not assess the reliability and validity of the confidence teaching measure and satisfaction measure. In future studies, we plan to address this by using validated questionnaires.

For future research, we would like to include survey measures to assess resident outcomes including quality of life and physical function. We would like to also include items that are not self-reported but are direct assessments of patient health outcomes that can be obtained from health records such as the minimum data set. Furthermore, for future studies we are interested in measuring the cost of a training program such as the one implemented in this study and to conduct a cost-benefit analysis.

## Conclusions and implications

We developed and refined a remote training program for PLIÉ-CLC and successfully trained multi-disciplinary CLC staff participants. Participants reported high satisfaction from the training and noted positive mood and health benefits in the residents from the PLIÉ classes. This remote training program will enable effective scaling and dissemination of PLIÈ-CLC at a national level.

## Electronic supplementary material

Below is the link to the electronic supplementary material.

Supplementary Material 1

## Data Availability

The datasets used and/or analyzed during the current study are available from the corresponding author on reasonable request.
