# Supplementary material for: Implementing a new multidisciplinary, remote, dementia staff training program for Veterans affairs nursing homes
Source: BMC Health Serv Res. 2024 Oct 3;24:1178. doi: 10.1186/s12913-024-11464-4 (PMC11450980; doi:10.1186/s12913-024-11464-4)
Supplement: Supplementary file 1 — Supplementary Material 1 [file 12913_2024_11464_MOESM1_ESM.docx]

**Supplementary File 1. Summary of PDSA Cycles’**

**APPENDIX**

Summary of PDSA Cycles’

(Should be included after PDSA Cycles section in “Results”)

| **Cycle** | **Plan** | **Do** | **Study** | **Act** |
| --- | --- | --- | --- | --- |
| **1** | Test the use of Microsoft Forms by administering a survey to obtain background information on PLIÉ staff participants | A link to a Forms survey was provided at the end of Orientation. The survey asked about the participant’s CLC role, their reason for their taking the PLIÉ training program, past experiences in working with individuals with dementia, and demographics (Table 1). In addition, it asked participants to rate their confidence teaching 8 PLIÉ principles on a scale from 0 (no confidence) to 10 (complete confidence). This Forms survey was also emailed to those who did not attend the live Orientation. This survey was also re-administered at the end of the training to examine change in confidence. | On the survey, participants described reasons for participating in the PLIÉ program with overarching themes that the participants want to better care for and engage Veterans with dementia. One said their goal was to learn new and “non-pharmaceutical” interventions and treatments to help patients with dementia. Another said they were hoping to learn how to “[meet] the behavioral challenges [of] people with Dementia”. A third individual said they wanted to “[explore] the many ways we can provide avenues for expression and providing a pathway to improved quality of life.” The average baseline confidence score for PLIÉ principles was 6.6 (SD: 0.8, Table 4). | Forms survey was effective for collecting feedback for those who attend live sessions.  Alternatives needed for those who do not attend live. |
| **2** | Obtain feedback on Module 1 didactic session | The Microsoft Forms survey asked the participants how likely they were to apply the PLIÉ principles with residents, skills and observations about themselves gained from the session, and suggestions for improving future training sessions. | All 11 of the trainees who participated live in the Module 1 didactic training completed the feedback survey; however, only 2 of 3 individuals who did not attend the live training reviewed the module content and completed the survey. Twelve out of 13 of those who completed the survey stated that they were “very likely” or “moderately likely” to use what they learned in the module in their work with residents. Similarly, 9 out of 13 trainees said what they learned in module 1 was “very likely” or “moderately likely" to change the way they work with residents. When asked “During today’s PLIÉ training session, what did you notice about yourself?” 10 out of 13 respondents discussed how the training enabled them to feel calmer or focus on their breath or the present moment. | For those unable to attend the live didactic sessions to learn the PLIÉ principles, a 10-minute video recording demonstrating key movements as a supplement to full-length class video recordings was created and sent. In addition, the email included links to training materials. |
| **3** | Test whether creating a 10-minute didactic video recording for Module 2 would encourage trainees who did not attend the live training to review didactic content and complete the feedback survey | A 10-minute Module 2 didactic video recording and link to the feedback survey was emailed to non-live attendees. The survey was a slightly edited version of PDSA Cycle 2 survey. The questions asked if the participants watched the video of exercises, if they found it helpful and if so, how much so, and if they used the additional resources, which included a 1-page handout summary about the module, the 10-minute video, the full Module 2 video, and the training PowerPoint slides. | Four people did not attend the live session and were sent links to the 10-minute video recording and survey, of whom two responded: one had watched full video, brief video, slides, and handout and found all “very useful”, and the other individual found the brief video “moderately useful” and the handout a “little useful” and did not review other content. | Two surveys were developed – one for those who attend the Module 3 live didactic session and another for those who do not. |
| **4** | Assess what materials are most helpful and are being used for both individuals attending the live PLIÉ-CLC training live and people unable to attend live sessions. | We sent out one feedback survey for those who attended Module 3 live and another feedback survey for those who did not attend the didactic session.  Survey questions for non-live attendees included those about why participant could not attend the session live, the best contact method, options used for module training and how helpful these options were. Survey questions for live attendees included asking how helpful each of the program elements were. | Nine out of 9 participants completed the survey for live attendees. Three of the 5 non-live participant attendees completed the survey after receiving an email and Teams message reminder. Nine out of 12 survey respondents, including the responses from the live and non-live attendees, stated that the experiential session was “very helpful”, while 3 individuals said it was “moderately helpful.” | The experiential component of the training was most helpful. Even with multiple reminders, not all the participants filled out surveys. This cycle demonstrated the importance of continuing to use multiple strategies to encourage those who do not attend live to review content and complete the surveys. |
| 5 | Determine what PLIÉ-CLC principles are being practiced and in what formats. This cycle combined information from 2 weeks and 2 modules (4 and 5), as the survey questions for both weeks were similar. | We distributed a survey about participants’ experiences using PLIÉ-CLC with residents and in their daily life. The surveys included questions about how often participants used PLIÉ in their personal and professional lives the last week and which principles they used, as well as questions on how frequently they used PLIÉ principles in their clinical encounters with residents. Other questions included free response observations about the impact of PLIÉ on the residents, the senior instructor, and themselves. | In the first week, nine of 12 survey respondents (75%) stated they used the PLIÉ principles “Sense & Breathe” and eight of 12 (67%) used “Go Slow” in the past week. In this cycle’s second week, eight of nine (89%) survey respondents said that they used PLIÉ-CLC in their personal lives one or more times in the past week. Additionally, six out of nine (67%) survey respondents in the cycle’s second week stated that they used PLIÉ principles with the Veterans at least once in the past week. The majority of survey respondents (89%) noticed the senior instructor’s gentle, individualized, non-judgmental and enthusiastic engagement with residents during facilitation of the experiential sessions. | It was concluded that further examples and instruction on how to integrate PLIÉ-CLC in daily interactions with residents as well as for staff’s personal lives were needed. |
| 6 | Test what PLIÉ-CLC principles are being practiced and in what formats | We encouraged participants to self-reflect on how they might incorporate PLIÉ principles into daily practice. We distributed a survey asking how staff planned to use PLIÉ in their daily interactions with residents and personal lives. The survey included questions how participants can use PLIÉ movements and themes in their daily encounters with veterans and in their personal lives in the upcoming week. The last question asked how the team could help the staff use PLIÉ more often with residents and themselves. | When asked how they could use PLIÉ movements and themes in their daily encounters with Veterans in the following week, five of 12 said they would use elements from the principle “Go Slow” with the Veterans. Additionally, five of 12 said they would use breathing or body awareness exercises inspired by the principle “Sense and Breathe” with the Veterans. When asked how could use PLIÉ movements and themes in their personal lives in the upcoming week, most respondents (67%) said they would consciously breathe throughout the day to center themselves. | In a future cycle, it was determined that it would be informative to ask what the staff participants did to incorporate PLIÉ in their daily interactions with residents and their personal lives. |
| 7 | Assess whether staff participants increased use of PLIÉ principles following the self-reflection exercise. | A survey was sent asking what staff did to use PLIÉ in their daily interactions with residents and in personal lives. Survey questions included those about how often participants used PLIÉ in their daily interactions with Veterans and in their personal life. The survey also asked for examples of these uses. | Eight out of nine survey respondents (89%) stated that they used PLIÉ in their daily interactions with Veterans at least once in the past week, of whom five (56%) used PLIÉ with their Veterans at least once every few days. The theme of “Go Slow,” such as being patient with Veterans and talking more slowly around them, was the most common PLIÉ principle used. Eight of nine survey respondents (89%) stated that they used PLIÉ in their daily life at least once in the past week: two used PLIÉ multiple times every day, one said about once a day, four said once every few days, and one said once every week. Seven participants described being mindful of their breath as examples of using PLIÉ in their daily lives. | This PDSA cycle demonstrated that asking participants explicitly to think about how they might incorporate PLIÉ principles into their daily interactions with residents in the previous cycle resulted in an increase in use of PLIÉ principles from 67% of participants using PLIÉ principles with residents in cycle 5 to 89% of participants incorporating the principles with residents in the current cycle. It was determined that optimal strategies to support maintenance of PLIÉ classes after the classes end in future cycles were needed to be asked about. |
| 8 | Determine what resources would be needed to support maintenance of PLIÉ classes after training completion and get feedback about future certification standards | The survey for this cycle asked participants for their ideas on how to implement PLIÉ classes on an ongoing basis and new resources that might help instructors feel comfortable leading or facilitating PLIÉ classes. Other survey questions include asking how adequate certain training activities would be to certify someone as a PLIÉ instructor. | Survey respondents reported that individual class teaching plans and a library of short videos would be the most helpful and flash cards with PLIÉ principles and themes would be least helpful. For activities that staff participants believed would be adequate to certify someone as a PLIÉ instructor, seven of the eight respondents agreed that it would be “Definitely Adequate” to “Participate in 6 to 8-session didactic + experiential program, including a test trainees would have to pass and leading a class with instructor observation.” | It was concluded that it would be beneficial to create and share resources that trainees identified as being most helpful.  The team also learned that it would be useful to develop a certification test to confirm knowledge of PLIÉ principles for future trainees. |
| 9 | Assess the changes in measures of confidence in those who completed the training and ask standard evaluation questions of both trainees and residents | A staff satisfaction survey was sent to ask the trainees about their experiences during the PLIÉ staff training program. The survey included questions about participants’ confidence levels doing certain PLIÉ activities with a group of residents with dementia. The next sets of questions asked about participants’ emotions during the training, their satisfaction levels, favorite and least favorite parts about the training program, and changes observed in themselves and residents. Other questions include asking how having VA Provider iPad affected staff members’ ability to participate in the PLIÉ-CLC staff training program and their ability to deliver care to veterans. | The average confidence scores for the 10 individuals who completed both the pre- and post-surveys declined slightly from before the training to after the training (from 6.6 ± 0.77 to 6.4 ± 0.81) (Table 4). Additionally, statistically significant decreases were reported in participants’ confidence levels to "Use movement sequences to help residents with dementia maintain function" (p = 0.04) and to “Use music and rhythm to support positive emotions" (p = 0.007).  When asked how having a VA Provider iPad affected their ability to participate in the PLIÉ-CLC staff training program, 90.9% (10/11) of the respondents stated that the iPad was helpful and positively affected their ability to participate in the training. At the end of the training, 100% (11/11) of respondents rated their overall satisfaction with the training as “very good” or “excellent.” The 11 participants who completed the survey stated that the PLIÉ training affected at least 80 residents. | Moving forward for Aim 3, the team learned that they could include portions of the training that were helpful to the participants such as the experiential and didactic elements of the sessions. The team can continue to incorporate the iPad into the training as well as continue to emphasize incorporating PLIÉ principles into trainees’ everyday lives to become comfortable and familiar with the program. |

**Supplementary File 2. Post-Training Survey Measures**

**Staff Satisfaction Survey**

| 1. How confident or certain that you can do these activities with a group of residents with dementia, on a scale from 1(Cannot do at all) to 7 (Certain can do)?    1. Create an environment to support joy and ease    2. Use breathing & body awareness to orient residents to the present moment    3. Move and talk slowly to give residents time to process and respond    4. Use movement sequences to help residents with dementia maintain function    5. Use music and rhythm to support positive emotions    6. Treat residents with patience, respect, & dignity    7. Support social engagement & emotional connection between residents    8. Build community between residents, staff, and family members 2. When participating in the PLIÉ-CLC staff training program, how often do you have these feelings?    1. I learn new skills for interacting with residents    2. I feel closer to residents    3. I feel that I am part of a team    4. I feel energized    5. I feel relaxed    6. I enjoy my work       1. Rarely       2. Sometimes       3. Often       4. Mostly 3. How would you rate your overall satisfaction with the PLIÉ-CLC staff training program?    1. Poor    2. Fair    3. Good    4. Very Good    5. Excellent 4. What do you like most about the PLIÉ-CLC staff training program? 5. What do you like least about the PLIÉ-CLC staff training program? 6. What changes have you noticed in yourself as a result of participating in the PLIÉ-CLC staff training program? 7. What changes have you noticed in residents as a result of your participation in the PLIÉ-CLC staff training program? 8. Is there anything else that you would like to say about your experience with the PLIÉ-CLC staff training program?   VA Provider iPads   1. How has having a VA Provider iPad affected your ability to participate in the PLIÉ-CLC staff training program? 2. Outside of PLIÉ, how has having a VA Provider iPad improved your ability to deliver care to Veterans? 3. How many Veterans have you used the VA Provider iPad with to provide any type of care, including PLIÉ? |
| --- |
| 1. Resident’s Name 2. These questions are about how often you feel certain ways in the PLIÉ classes. For each question, please tell me if you feel that way rarely, sometimes, often, or mostly.    1. I feel that I belong    2. I feel that I am accepted    3. I feel that my problems are similar to other veterans    4. I feel energized    5. I feel relaxed    6. I enjoy being together with a group of people like me       1. Rarely       2. Sometimes       3. Often       4. Mostly 3. How would you rate your overall satisfaction with the PLIÉ program?    1. Poor    2. Fair    3. Good    4. Very good    5. Excellent 4. What do you like most about the PLIÉ classes? 5. What do you like least about the PLIÉ classes? 6. What changes have you noticed in yourself because of the PLIÉ classes? 7. What changes have you noticed in others in the class because of the PLIÉ classes? 8. Is there anything else that you would like to say about your experience with PLIÉ? |

**Post-training focus group discussion guide Interview Script**

The goals for today’s discussion are to understand:

1. What worked well and less well for you during the training process; and
2. Suggestions to improve the training process

**Didactic sessions**

First, I’d love to hear about the didactic sessions and **what worked well** for you. Who’d like to start us off? What are some things that **didn’t work well** for you?

Follow-up prompts: Teams channel; Module slides – learning themes, principles, movements; short videos with movements; 1pg handouts]

**Experiential sessions –**

Switching gears to the experiential sessions with residents, we have the same questions -- **what worked well**? What are some things that **didn’t work well**?

[Follow-up prompts: technology, sensory tools, selecting residents, PLIE note template]

**Materials & Resources**

We’d love to hear your thoughts on the training materials and resources provided as part of the PLIÉ training.

[Follow up on any materials/resources not already discussed]

- iPads
- PLIE Instructor Manual
- Resource Guide
- Short videos (movements)
- Recordings of Modules (didactics, experientials)
- Monthly lunch & learn

**Suggestions for implementing at future sites**

As we expand to additional study sites and other CLCs, we’d love to hear your insights and suggestions for how to implement staff training based on your experiences.
